# Outpatient, dental care of adult vulnerable patients under general anaesthesia—a retrospective evaluation of need for treatment and dental follow-up care

**DOI:** 10.1007/s00784-020-03564-2

**Published:** 2020-09-15

**Authors:** Julia Jockusch, Werner Hopfenmüller, Ronald Ettinger, Ina Nitschke

**Affiliations:** 1 Clinic of General, Special Care and Geriatric Dentistry, Center of Dental Medicine, University of Zurich, Plattenstrasse 11, 8032 Zurich, Switzerland; 2 Institute of Biometry and Clinical Epidemiology, Charité – Universitätsmedizin Berlin, corporate member of Freie Universität Berlin, Humboldt-Universität zu Berlin, and Berlin Institute of Health, Charitéplatz 1, 10117 Berlin, Germany; 3 Department of Prosthodontics, University of Iowa, Iowa City, IA 52242 USA; 4 Department of Prosthodontics and Materials Science, Gerodontology Section, Universitätsklinikum Leipzig AöR, Leipzig, Germany

**Keywords:** General anaesthesia, Dementia, Dental phobias, Addiction, Psychiatric disorder, Disabilities, Dental treatment

## Abstract

**Objectives:**

To analyse the treatment needs of patients who had received dental treatment under GA and the effectiveness of the treatment provided.

**Materials and methods:**

Retrospective chart analysis of adult at risk and vulnerable patients requiring dental treatment under GA (2007–2017). Outcome variables were indications for GA, DMF/T, and type of treatment, failure rates of treated teeth, emergencies and recall intervals after GA.

**Results:**

Four hundred fourteen subjects (median age 42 years, range 18–93 years) were assigned to four groups (people with disabilities (pwdis), dementias (pwd), dental phobias (pwph), and addictions/psychosocial disorders (pwapd)) and attended the pre-GA assessment. Of these, 247 subjects (median 37 years, range 18–93 years) were treated under GA, mostly pwdis (*n* = 154, 69.7%). The main indication for treatment under GA was suspicion of pain (*n* = 178, 72.1%). Pwd had the highest degree of restoration (46.7%), DMF/T value (23.8), and most missing teeth (5.8). Pwapd had the most decayed teeth (12.9). There was a 12-month recall augmented by 2–4 oral hygiene sessions depending on compliance. The failure rate of all treated teeth was 4%. Two dental emergencies were reported for patients who received a GA.

**Conclusions:**

Dental treatment need was high for adult vulnerable people. The diagnostic groups differed mainly in their subjective reason for need of a GA, their DMF/T, treatment needs and type of treatments performed. Failure and dental emergency rates after GA were low in spite of a recall interval of 12 months.

**Clinical Relevance:**

Regular annual recalls could avoid dental emergencies in patients requiring treatment under GA.

## Introduction

The dental treatment of adult vulnerable patients such as persons with disabilities (pwdis), persons with dementia (pwd), and those with addiction and psychosocial disorders (pwapd) or dental phobias (pwph) is a great challenge for the dentist and his/her team. The problem is exacerbated due to the limited or non-existent health literacy of the patients and their families which leads to a limited cooperation. Often, the underlying disease results in the patients’ reduced ability to maintain daily oral hygiene either independently or with the help of third parties. Poor oral hygiene leads to oral diseases (caries, periodontitis, etc.), which can result in a greater need for treatment [1, 2]. In addition, vulnerable patients such as those with dementia or disabilities are often unable to recognise and/or describe their dental problems or pain. Effective communication with many patients may not be possible and makes targeted dental therapy difficult [3, 4]. In addition, diagnosis and treatment of the oral problems may be difficult or not at all possible in a conventional dental setting due to a lack of patient cooperation, despite the skill and training of the dentist and his/her team. In order to be able to provide these patients with an adequate standard of dental care, it is often necessary to utilise sedation under medical supervision and/or general anaesthesia (GA) [5–8]. The difficulty for the dentist is that for many of these patients, the decision to proceed with dental treatment under GA must be made without knowing in advance what their oral problem is [9, 10]. Primary indicators for dental treatment under GA are the lack or absence of patient cooperation due to anxiety, mental disabilities or other impairments [8, 11–16]. According to the Helsinki Public Dental Service, non-compliance (65%), dental phobias (37%) and urgent need for dental treatment (26%) were the main reasons for carrying out dental treatment under GA [17–21].

There are several studies which have investigated the outcomes of dental treatment using GA in healthy children [22–25]. However, a review of the literature provides little information on the treatment of adult patients with special needs under GA [26, 27]. Dental treatment using GA can improve patient safety and better outcomes because treatment planning as well as interventions performed carefully and correctly treatment under GA (e.g. restorations using rubber dam, etc.) are possible [28]. A GA should be considered for patients for whom dental treatment in the dental chair is not possible even when using other adjuncts such as sedation or nitrous oxide. However, in a previous review study using GA, there was a tendency to extract teeth rather than using more conservative measures (e.g. endodontics) in order to avoid possible failures and complications [29]. Nevertheless, in some patients, it was possible to complete a large number of restorative procedures under GA [14, 30].

If a dentist is unable to adequately examine the patient, then a diagnosis in advance of treatment may be limited or not possible; therefore, treatment under GA can only be planned to a limited extent. This in turn increases the need for the dentist and his/her team to comprehensively treat the patient while using GA. One of the advantages of outpatient GA is that many teeth can be treated in one session. This is associated with a reduction in stress for the patient and his/her accompanying person as well as a reduction in cost of care and transport [10]. Nevertheless, for vulnerable persons, there are possible anaesthetic risks with complications, and these should not be underestimated [31].

The aim of this retrospective study was (a) to characterise the group of adult vulnerable patients attending a pre-GA assessment at a specialised dental clinic, (b) to analyse the dental treatment needs of adult vulnerable patients treated under GA and (c) to evaluate the effectiveness of the treatment which was assessed by monitoring the outcomes, which are the type of restorations, the changes in the DMFT index over time, failure rates of treated teeth, changes in the indication for GA over time, occurrence of emergencies after treatment under GA and dental recalls and/or further treatments under GA.

## Material and methods

This study was a retrospective review of the charts of vulnerable dental patients (adults ≥ 18 years) at a specialised dental clinic in Switzerland from 2007 to 2017. All charts of patients who attended the pre-GA assessment and were diagnosed to need dental treatment under GA (TIVA —total intravenous anaesthesia) were included in the evaluation. The proportion of patients who completed treatment under GA related to all patients who attended the pre-GA assessment were also assessed (all patients who received general anaesthesia).

This clinic where this study was conducted is in the dental treatment of the elderly and adult people with disabilities or vulnerable patients in general specialized. These patients are mostly living in the canton of Zurich but are not limited to it. The clinic is a cantonal service provider and reaches a large number of patients through its contacts with the cantonal nursing homes and institutions for the disabled. The clinic has a mobile care concept (mobiDent™) in addition to an inpatient treatment facility [32]. Nevertheless, access to dental treatment for these vulnerable patients is not limited to the use of the services of this specialised clinic. All dentists in the canton of Zurich and throughout Switzerland can treat vulnerable patients, including using GA. On average, the clinic has carried out about 5000 treatment sessions for 3000 patients per year.

The subjects included in this study were divided into four groups according to their main medical diagnosis which were persons with disabilities (pwdis), persons with dementia (pwd), persons with addictions and psychosocial disorders (pwapd) and persons with dental phobias (pwph).

In addition to sociodemographic data (age, sex, medical risk factors, oral functional capacity (OFC)) [33], data on dental treatment carried out under GA were also recorded as a classification by group such as indication for treatment under GA, DMF/T index, therapy performed, duration of therapy, aftercare and failures.

Oral functional capacity (OFC) is shown in Table 1 and was used to assess patients from the multifactorial perspective of a specialist geriatric dentist considering a variety of aspects influencing on how to treat the patient. OFC is used to assess patients in three parameters: therapeutic capability, oral hygiene ability and self-responsibility. The worst evaluation in one of the three parameters determined the patient’s resilience capacity level (RCL). [33] (Table 1)

**Table 1:** Description of the oral functional capacity consisting of four resilience capacity levels (RCL 1–RCL 4) and three parameters (therapeutic capability, oral hygiene ability, self-responsibility) [33]. The parameter with the worst evaluation was used to classify the subjects in one of the resilience capacity levels (RCL)

Resilience capacity level (RCL)	Therapeutic capability	Oral hygiene ability	Self-responsibility
RCL 1 Normal	Normal	Normal	Normal
RCL 2 Slightly reduced	Slightly reduced	Slightly reduced	Normal
RCL 3 Greatly reduced	Greatly reduced	Greatly reduced	Reduced
RCL 4 No resilience	None	None	None

The DMF/T index (D, decayed; M, missing; F, filled; T, teeth) is described as a measure of caries experience. [34]. The World Health Organization (WHO) criteria were used for decayed and filled teeth. Early stages of caries as well as stages of caries that precede cavitation were excluded from being recorded as decayed teeth. Older people often do not know why they had lost a tooth many years ago. Therefore, it is difficult to differentiate whether a tooth was lost due to caries or periodontal disease or trauma. Thus, the DMF/T index has not been used as an accurate measure of caries experience in gerodontology, but rather as an epidemiological description for elderly people [34, 35]. Therefore, missing teeth (MT) in this analysis include teeth missing due to caries, periodontitis or trauma. The DMF/T index is related to 28 teeth.

The degree of restoration (in percent, %) was calculated from components of the DMF/T index and was calculated as follows: (F/D + F) × 100.

Periodontitis and gingivitis were defined by using the Periodontal Screening Index (PSI) [36], which was collected per sextant (code 0, healthy; code 1, gingivitis without calculus/plaque and without defective restoration margins; code 2, gingivitis with calculus and/or plaque and/or defective restoration margins; code 3, moderate periodontitis; code 4, severe periodontitis).

On follow-up, failure rates/error rates refer to biological complications of already treated teeth of the patients who had received treatment under GA. These include secondary caries of previously restored teeth, fractures (spontaneous or due to trauma) as well as apical periodontitis.

Statistical evaluation was purely descriptive using SPSS Version 23 [37].

The study was approved by the data protection officer of the canton of Zurich and classified as not requiring approval by the responsible cantonal Ethics Committee Zurich and was therefore approved (ID: Req-2018-00597).

## Results

During the 10-year observation period, 456 patients were registered during the first pre-GA assessment. Of these, 414 patients (100%) appeared for their appointments and were included in this analysis as 29 were removed from the study because they failed the first appointment, and another 13 were removed because they were under 18 years of age.

**Fig. 1:**
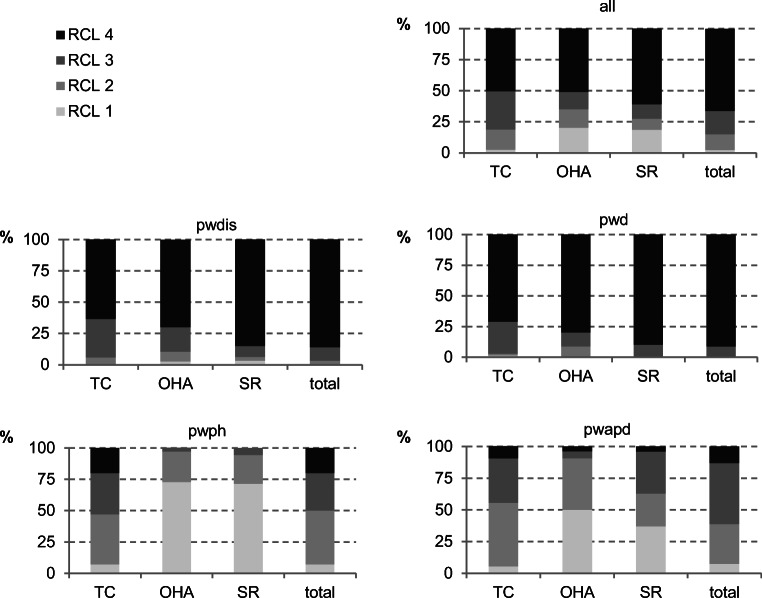
Oral functional capacity of all the subjects by their main diagnostic groups is shown here (TC, therapeutic capability; OHA, oral hygiene ability; SR, self-responsibility; RCL, resilience capacity level; RCL 1, normal; RCL 2, slightly reduced; RCL 3, greatly reduced; RCL 4, no resilience) (all patients *n* = 414; people with disabilities (pwdis) *n* = 221; people with dementia (pwd) *n* = 69; people with dental phobia (pwph) *n* = 70; people with addiction and psychosocial disorders (pwapd) *n* = 54)

An assessment of oral functional capacity found that pwdis and pwd were less resilient. However, for pwph or pwapd, the treatment for half of these subjects was assessed as normal or slightly reduced. The assessment of the subject’s oral hygiene ability and self-responsibility were also classified as normal or slightly reduced (Fig. 1).

### General anaesthesia

Of all the subjects who attended for the pre-GA assessment (*n* = 414, 100%), those with pwdis received the most dental treatment under GA (*n* = 154, 69.7%). For pwapd, half of them (*n* = 27, 50.0%) were treated under GA, compared with less than half of the pwd (*n* = 69, 46.4%) or pwph (*n* = 70, 48.6%) (Table 2).

**Table 2:** Total number of subjects receiving a treatment under GA* as well as the dental indications for each subjects’ first treatment under GA as well as number of GA’s** performed per subject for all subjects and stratified according to their primary diagnosis

	All subjects	pwdis	pwd	pwph	pwapd
	[*n*/%]	[*n*/%]	[*n*/%]	[*n/*%]	[*n*/%]
Total number of subjects receiving treatment under GA after the first pre-GA assessment (all *n* = 414) *[*n*/%]	247/59.7	154/69.7	32/46.4	34/48.6	27/50.0
Age at the first GA in years					
Mean	41.1	35.9	75.7	33.6	39.1
SD ±	18.6	14.0	10.7	11.2	10.7
Median	37	34.5	78	31	39
Range	18–93	18–70	58–93	19–59	23–65
Sex					
Male	140/56.7	95/61.7	6/18.7	18/52.9	21/77.8
Female	107/43.3	59/38.3	26/81.3	16/47.1	6/22.2
Dental indication for every subject at the first GA (Decision was made in the first pre-GA assessment.)					
(Suspicion of) pain	*178/72.1*	*97/63.0*	*27/84.4*	*28/82.4*	*26/96.3*
Dental check-up	51/20.6	47/30.5	4/12.5	0/0	0/0
Referral from medical practitioner	4/1.6	2/1.3	0/0	1/2.9	1/3.7
Desire for oral rehabilitation	5/2.1	1/0.7	0/0	4/11.8	0/0
Referral from other dentists	9/3.6	7/4.5	1/3.1	1/2.9	0/0
Observation period in years					
Mean	3.0	3.4	1.4	2.8	3.0
SD ±	2.7	2.8	2.1	2.5	2.3
Number of GAs per subject** (*n*/% related to the total number of subjects receiving treatment under GA after the first pre-GA assessment (*n* = 256))	(*n* = 247)	(*n* = 154)	(*n* = 32)	(*n* = 34)	(*n* = 27)
One	193/78.1	*113/73.4*	*28/87.5*	*28/82.4*	*24/88.9*
Two	45/18.3	33/21–4	3/9.4	6/17.6	3/11.1
Three or more	9/3.6	8/5.2	1/3.1	0/0	0/0
Total number of GAs in all subjects receiving GA during the observation period [n]	312	205	37	40	30

*Total number of subjects receiving treatment under GA for whom the decision was made at the first pre-GA assessment. Number is related to the number of subjects who were seen at the first pre-GA assessments in total (*n* = 414) or per group (pwdis *n* = 221, pwd *n* = 69, pwph *n* = 70, pwapd *n* = 54)

**Total number of GA’s per subject during the observation period of 10 years related to the number of subjects for whom an indication for a treatment under GA was made at the first pre-GA assessment (total *n* = 247, pwdis *n* = 154, pwd *n* = 32, pwph *n* = 34, pwapd *n* = 27)

Of all patients treated under GA (*n* = 247, 59.7%), 87 patients (35.2%) had risk factors/modifying factors that complicated treatment and needed to be considered prior to dental treatment. The most frequent risk factor was drug and/or alcohol abuse (57, 65.5%). Furthermore, 16 patients (18.4%) were prescribed anticoagulants or had an infectious disease (*n* = 15, 17.2%) which made dental treatment more difficult. Four patients (4.6%) were taking bisphosphonates and two patients (2.3%) had had head and neck radiation.

Most subjects treated under GA during the observation period received only one or two treatments under GA. Three or more GAs where carried out only for pwdis and/or pwd during the 10-year observation period. A total of 247 patients (59.7%) for whom the decision for a treatment under GA was made in the first pre-GA assessment (total number of patients in the pre-GA assessment *n* = 414, 100%) received treatment under GA. Approximately every 5th patient received two treatments under GA (*n* = 45, 18.3% related to all patients receiving treatment under GA (*n* = 247)); only 9 patients (3.6% related to all patients receiving treatment under GA (*n* = 247)) received three or more treatments under GA. The number of GAs per patient resulted in a total of 312 completed GAs during the observation period (Table 2). The GA’s were predominantly outpatient procedures (*n* = 300; 96.2%). Only one patient had chairside sedation (0.3%); four patients had sedation under GA conditions (1.3%). Inpatient admission with GA was carried out for seven treatments in seven patients (2.2%).

The main reason for an application for a treatment under GA in pwdis was the wish for a dental check-up (*n* = 95, 43% of 221 patients who attended the pre-GA assessment in this group), whereas those with pwph often indicated a need for oral rehabilitation (*n* = 39, 55.8% of 70 patients who attended the pre-GA assessment in this group). However, the indication for GA in these two groups described by the dentist of the special department was usually due to the suspicion of pain (Table 2). Only with pwd did the pre-treatment reason for need for treatment under GA during the enrolment assessment (suspicion of pain, *n* = 52, 75.4% of 69 patients who attended the pre-GA assessment in this group) and dentists’ assessment almost agree (Table 2).

The first GAs lasted a median of 200 min (GA 1: range 40–540 min.). The shortest GA treatments were for pwdis (median: 180 min, range 60–420 min.) and pwd (median: 180 min, range 40–350 min.) and the longest for pwapd (median: 300 min; range 60–540 min.). The duration of GA was shorter during follow-up GAs (2nd GA: median 180 min, range 50–395 min; the 3rd GA had a median of 150 min; range 120–210 min). The mean interval between the first and second GA was 3.1 years (SD ± 2.2 years), and between the second and third GA, it was 3.2 years (SD ± 1.1 years).

### Dental findings and therapies

**Fig. 2:**
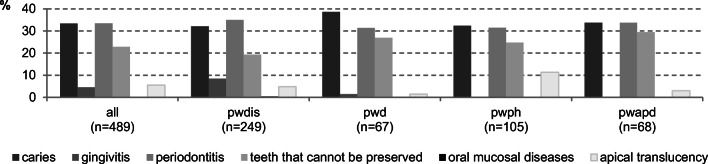
Dental diagnosis at the time of the first GA for all subjects and by their diagnostic groups (%) (multiple responses were possible) (people with disabilities (pwdis); people with dementia (pwd); people with dental phobia (pwph); people with addiction and psychosocial disorders (pwapd))

At the time of the first GA, the main dental findings were caries, periodontitis and unrestorable teeth. Those with pwph, pwapd and pwd had more teeth that could not be restored than pwdis. Periodontal diseases were common in all diagnostic groups. Periapical lesions and changes in the oral mucosa were rare in all subjects (Fig. 2).

The DMF/T value was highest among pwd at 23.8. Most carious teeth were found in pwapd (DT 12.9), pwph (DT 10.9) and pwd (DT 9.6). The pwd (MT 5.8) had the most missing teeth and the highest number of restored teeth before treatment under GA (46.7% degree of restoration) followed by pwdis (41.7% degree of restoration). The pwapd had the lowest percentage of restorations at 7.2%. The calculation for degree of restoration is shown in the “Materials and methods” section (Table 3).

**Table 3:** DMF/T index and individual values for all subjects by the main diagnostic groups before and after the first GA (*n* is depending on the availability of complete oral findings in the charts). (DMFT index: D, decayed; M, missing; F, filled; T, teeth; related to 28 teeth) (people with disabilities (pwdis); people with dementia (pwd); people with dental phobia (pwph); people with addiction and psychosocial disorders (pwapd))

	Age (years)	DMF/T		DT		MT		FT		Degree of restoration [%]	
	Mean ± SD	Pre	Post	Pre	Post	Pre	Post	Pre	Post	Pre	Post
All subjects (n_pre_ = 242/n_post_ = 236)	40.2 ± 18.9	12.4	13.2	6.3	0.4	3.0	6.9	3.1	5.9	32.3	93.7
pwdis (n_pre_ = 151/n_post_ = 146)	34.7 ± 14.4	8.1	9.0	3.5	0.1	2.1	4.1	2.5	4.8	41.7	98.0
pwd (n_pre_ = 31/n_post_ = 30)	75.7 ± 10.7	23.8	24.6	9.6	0.9	5.8	15.0	8.4	8.7	46.7	90.6
pwph (n_pre_ = 34/n_post_ = 34)	33.6 ± 11.2	16.9	17.4	10.9	1.1	3.4	8.4	2.6	7.9	19.3	87.8
pwapd (n_pre_ = 26/n_post_ = 26)	39.6 ± 10.6	18.4	18.8	12.9	0.7	4.5	11.4	1.0	6.7	7.2	90.5

The type of dental treatment provided included diagnostic imaging with radiographs, dental hygiene, surgical treatment (*n* = 1141; 54.2%), restoration of teeth (*n* = 905; 41.9%) and endodontic treatment (*n* = 60, 2.8%). Most extractions (*n* = 366) were required for the pwds (*n* = 289, 79.0%). For all other diagnostic groups, the ratio between restorative care and surgical treatment was almost evenly distributed. However, for the pwapds, the predominant treatment was extractions (*n* = 219, 58.2%) (Table 4). The extraction of all remaining teeth in the maxilla alone was required for five subjects (2.0%) and in the mandible for one subject (0.4%). In 15 subjects, all remaining teeth in both arches were extracted (6.0%); at the same time, 10 of those subjects had dementia. Maxillary teeth and mandibular molars were those most frequently requiring either extraction or restorative treatment. Endodontic treatment was predominantly carried out in the maxillary anterior region and in the mandible for the canines and premolars.

**Table 4:** Number and type of teeth treated including failures and the further treatment for all subjects and according to their main diagnostic groups (*n*/%) (people with disabilities (pwdis); people with dementia (pwd); people with dental phobia (pwph); people with addiction and psychosocial disorders (pwapd))

		All subjects	pwdis	pwd	pwph	pwapd
		(n/%)	(n/%)	(n/%)	(n/%)	(n/%)
	Dental treatment under GA					
Number of treated teeth (total)		2106	850	366	514	376
Restored teeth	Related to total:	905/41.9	*442/52.0*	77/21.0	234/45.5	152/40.4
	*of this:*					
	Composite restoration	716/79.1	394/89.1	70/90.9	153/65.4	99/65.1
	Glass ionomer cement restoration	173/19.1	41/9.3	7/9.1	81/34.6	44/28.9
	Amalgam restorations	16/1.8	7/1.6	0/0	0/0	9/6.0
Extractions	Related to total	1141/54.2	389/45.8	*289/79.0*	244/47.5	219/58.2
Endodontic treatment	Related to total	60/2.8	19/2.2	0/0	*36/7.0*	5/1.4
Failures after treatment under GA						
Failures	*Related to:*					
	Number of treated teeth	84/4.0	43/5.1	5/1.4	19/3.7	17/4.5
	Number of restored teeth	84/9.3	43/9.7	5/6.5	19/8.1	17/11.2
Type of failure after treatment under GA						
Type of failure	Secondary caries	80/95.2	40/93.0	5/100	18/94.7	17/100
	Tooth fractures	3/3.6	3/7.0	0/0	0/0	0/0
	Apical periodontitis/lesions	1/1.2	0/0	0/0	1/5.3	0/0
Further therapy for teeth affected by failure						
	Extraction	26/31.0	10/23.3	3/60.0	3/15.8	10/58.8
	Endodontic treatment	1/1.1	0/0	0/0	1/5.3	0/0
	Restorative treatment	57/67.9	33/76.7	2/40.0	15/78.9	7/41.2

The failure rate for all treated teeth (*n* = 2106) was 4.0% (*n* = 84); for all restored teeth (*n* = 905), it was 9.3% (*n* = 84). The most common cause for failure was due to secondary caries (*n* = 80; 95.2%). Those teeth were either re-restored (*n* = 57, 67.9%) or extracted (*n* = 26, 31.0%) (Table 4).

### Additional evaluations of monitored outcomes—effectiveness of the treatment

#### Indications for GA

The indications for GA changed over time. The indication at the first GA was mainly based on the suspicion that the subject was in pain (*n* = 178, 72.1%). The indication to use GA for follow-up examinations increased during the observation period (indication for 1st GA: 20.6%; before 2nd GA 42.2%; before 3rd GA 62.5%).

#### Emergencies after treatment under GA

Among the 247 subjects who were observed for a mean of 3 years ± 2.7 years, there were two emergencies (0.1%) out of a total of 2106 treated teeth. Subjects who could have benefitted from a GA due to their dental problems but did not receive one because of health reasons either because it was too risky for them to be anaesthetised or because relatives/caregivers refused to give permission, however, did not experience any dental emergencies (abscesses, pain, etc.) during the observation period.

#### Dental recalls and further treatments under GA

Of all subjects (*n* = 414) who attended the pre-GA assessment, eight (1.9%) were not given any further appointments for a dental check-up/GA due to their non-compliance (e.g. extreme aggression against others or themselves under stress). Since the diagnosis for these eight subjects was based exclusively on observation without an oral examination, no further appointments were made at the clinic. The relatives and the nursing staff of these subjects were instructed on how to recognise the signs and symptoms of oral pain and how to help the patients with oral hygiene. In addition, eight subjects were not given oral hygiene appointments in the clinic but were given GA appointments for follow up visits.

All other subjects who were able to have professional dental cleaning in a dental chair done by a dental hygienist were recalled to see the dentist annually. At this appointment, the dentist together with the patient and/or the patient’s relatives/carers decided whether a GA was required for dental treatment separate from the necessary professional oral hygiene appointments.

In subjects who were not able to receive a professional dental cleaning in the dental chair, the dentist had to determine whether a recall examination for oral hygiene care was necessary. The dentist would then on an annual basis consult with the subject’s relatives at what time and under what conditions (sedation in dental clinic vs. GA) a recall for oral hygiene and/or dental check-up should take place.

An indication for further treatment under GA was considered if at least one of the following criteria was met: (a) injury to the face or the teeth due to violence or fall, (b) acute clinical findings (e.g. swelling, chipped teeth with exposed pulp), (c) diagnostic changes in character and/or behaviour of the subject that was not due to any other cause after a medical consultation (e.g. restlessness, aggression, which could be a sign that the subject was in pain), (d) if oral hygiene could not be carried out by the dental or nursing staff but the subject had inflammation and/or severe halitosis and there was a referral from the subject’s physician or another dentist to treat him or her.

After the pre-GA assessment (*n* = 414), 247 subjects (59.7%) received a GA either as an outpatient or inpatient depending on their risk for anaesthesia, their ability to pay for care and the consent from their legally responsible caregiver. Ten subjects (2.4%) received a median of three recalls (range 2–10 recalls, a total of 40 recalls for the 10 subjects) after the first appointment before they had an initial treatment under GA.

A second GA treatment was required for 45 of the 193 subjects who received a first GA. Of these, 17 subjects (37.8%) had no further recall appointments between the first and second GA treatment due to non-compliance or because a second GA was necessary to complete treatment. Forty subjects (88.9%) had a median of two recalls (range 1–15, total 156 recalls) between the first and second GA.

A third GA treatment was required for nine subjects. Of these, two subjects (22.2%) received no further recalls between the second and third GA treatment due to non-compliance. Seven subjects (77.8%) had a median four recalls (range 2–18, total 38 recalls) between the second and third GA.

## Discussion

In order to highlight differences between patients’ dental care needs, their indications for dental treatment under GA and the effectiveness of treatment, the charts of adult vulnerable patients who required dental treatment under GA during a period of 10 years were classified according to one of the four diagnostic groups, these were: people with disabilities (pwdis), people with dementia (pwd), people with addiction and psychosocial disorders (pwapd), and people with dental phobias (pwph). Generalisability of these findings is limited because this is a specialised clinic which has a mobile care program (mobiDent™) as well as an inpatient treatment facility [32]. This program enables pwdis and pwd who live or are cared for in institutions to be treated chairside and to avoid GA through a long-standing professional and familiar contact and close cooperation with their nurses/carers due to a regular prophylactic oral hygiene programme. Dental emergencies are dealt with more directly and become more manageable. Therefore, it must be assumed that pwd and pwdis in need of a GA will be underrepresented in this analysis.

Twenty-nine patients who had been identified as needing treatment but did not appear for the first pre-GA assessment because they either moved, died or received treatment under GA at another institution were not included in this analysis.

The pwdis were younger and therefore were treated more often over time under GA because it was required for oral health check-ups. The primary philosophy of care was to maintain their oral health and chewing function over the long term; this resulted in them having the highest rate of restorative treatment. The oral health and functional concerns were also important for pwd. Most of these subjects were very old and had multiple co-morbidities and polypharmacy and were seen infrequently. Therefore, dental treatment under GA was more restrictive. Their extraction rate was higher because at risk teeth were extracted to avoid the need for further GAs. The pwphs as well as the pwapds could often be treated at the chairside, mostly for prosthodontic needs, after GA. The help of a psychologist who specialised in the care of dental phobics reduced the need for GAs. If these subjects received more than one GA, it was usually due to the fact that their initial treatment needs were so high that the scheduled time for one GA was inadequate to complete the treatment required.

Often the loss of independence due to cognitive impairment was accompanied by a reduced ability or difficulty in maintaining personal oral hygiene. This in turn can result in an increased risk of more caries and periodontal disease [38]. This problem was clearly reflected in the numbers of carious and/or unrestorable teeth in this population. The DMF/T value of the subjects in the diagnostic groups was difficult to compare due to a broad age range. Taking the mean age of all patients as a basis in order to classify the DMF/T value, all patients had DMF/T values comparable with the findings in the largest dental epidemiological study in Germany (Fifth German Oral Health Study (DMS V)) [39]. It should be taken into account that DMS V did not include people with severe disabilities or dementia in the age group “younger adults” (DMS V: disease and care prevalences in younger adults (35- to 44-year-olds), *n* = 966, 90 study points nationwide, representative of the German population).

If the diagnostic groups are considered separately, it can be seen that the DMF/T values were lower for pwdis (DMF/T 8.4 compared with 11.2 for DMS V [39]). This can be explained by the fact that although the number DT was higher than in the representative comparison group, the FT was significantly lower due to the lower number of dental appointments (treatment only possible in GA). The opposite was true for the pwphs (DMF/T 17.6) and the pwapds (DMF/T 18.8): here the number of DT was higher due to the fact that the subjects had not consulted or been treated by a dentist so FT value was higher also [39]. For pwd, the DMF/T value was 23.2, which is similar to that of 75- to 100-year-olds with care needs in the DMS V (DMF/T 24.5) [40]. However, the DMS V study reported more restorations (66%) compared with subjects with pwd in this analysis. This is also reflected in a significantly higher DT and FT value. In this study, subjects with pwd had a high need for treatment measured by the DT value. At the same time, they had more restorative treatment and fewer teeth extracted. The reason for this may be that in Germany and Switzerland—due to different financing structures—different treatment protocols exist.

In this evaluation, a recall interval of 12 months was augmented by 2–4 oral hygiene sessions per year for subjects who accepted chairside treatment.

Regular dental recalls after GA are essential to re-instruct patients and their carers on how to perform daily oral hygiene [41] and how to detect pathological changes. The carers (professional and/or private) should be trained to perceive dental problems among those entrusted to their care, to be able to organise treatment, to perform daily oral hygiene supported by the prescription of high concentration fluoride toothpastes and also by paying attention to nutrition.

Recall intervals of 12 months with a dentist for subjects who cannot be treated chairside for professional oral hygiene treatments have been implemented. These intervals can only be achieved with very good communication and information transfer between the persons responsible for the (dental) medical, nursing and socio-educational care of vulnerable patients. Within this close cooperation, early signs of oral problems (e.g. pain, refusal to eat, restlessness) in vulnerable patients can be dealt with and if a necessary GA is required it can be planned. In this study, the analysis of patients who had a GA for dental treatment did not have any emergency problems such as abscesses during the observation period.

The introduction of recalls to improve behaviour in the dental chair has been discussed in the literature in order to reduce the number of GAs [42]. This concept may not apply to pwd and pwdis, since they have difficulties with accommodation to dental treatment.

The literature suggests that shorter follow-up intervals of 4–6 months [26] or even every 2 months [43] would be helpful. Due to limited clinic staff and spatial capacity as well as the high demand on carers of the disabled, these short follow-up intervals could not be utilised in our specialised clinic.

There are very few life-threatening odontogenic emergencies in dentistry. Even emergency situations such as an abscess can usually be well treated in European health systems. Even for vulnerable patients who do not have regular dental treatment, a short-term intervention can help such as the administration of antibiotics while dental treatment under GA is being organised. Therefore, the question arises to what extent should GAs be used for dental treatment? It is not clear how long the time span should be between GA treatments for patients who are uncooperative [44]. There is also no data in the literature on how safe it is for patients to receive repeated GA treatments [15].

The most common side effects of GA are memory impairment and limited ability to cope with intellectual tasks. Postoperative cognitive dysfunction (POCD) is a transient cognitive dysfunction which can occur after surgery and should be discussed with the patient/carers when GA is proposed for dental treatment. The course of dementia can be influenced by anaesthesia. Patients with Alzheimer’s dementia had more cognitive impairment 5–9 months after surgery [27]. This occurred especially in patients who had already been impaired preoperatively [27]. Alcohol [45] and drug abuse as well as the level of education [46–48] are also risk factors for the development of POCD. The risk also increases with age, as 41.4% of post-operative patients over 60 years of age who were discharged from hospital had POCD [45, 49]. Gas anaesthesia intensifies this phenomenon [50] while total intravenous anaesthesia (TIVA) has fewer side effects. Possible cognitive disorders can negatively influence both morbidity and mortality in geriatric patients. Deficits can therefore occur with a time delay and persist for a long time. [51–53]. Delirium needs to be distinguished from POCD.

Kline et al. (2012) using MRI examinations showed a decrease in grey matter, atrophic changes of the hippocampus and a relative increase in the volume of the lateral ventricles 5–9 months after surgery. Postoperative cognitive impairment was particularly common in patients with mild subclinical cognitive impairment prior to surgery. The difference between patients with and without surgery was not statistically significantly different because over time the dementia progressed. [27]

It may be expected that dental treatment under GA will increasingly be required for patients with multiple co-morbid problems and patients with disabilities, because they are living longer and becoming elderly. Inpatient treatment may be required to avoid complications such as patients needing intensive care after GA. The site for the dental treatment of vulnerable patient groups under GA should be in a hospital [15, 26, 54].

As a result of this review, the authors conclude that GA should only be used if it is indicated, not routinely and only if other methods (e.g. drug sedation, nitrous oxide, etc.) have failed. It is important that the risk to the patient’s health, the efforts for the caregiver and the associated costs of a GA are always taken into account [8, 15, 44].

## Conclusion

The dental treatment needs are high for adult vulnerable people who need treatment under GA. Suspected or confirmed pain or dental complaints were the main indication for treatment under GA. The diagnostic groups differed mainly in their main reason for seeking care, their dental treatment needs as well as the type of treatments performed and their DMF/T values. The aftercare/need for dental recall was determined by the patients’ compliance to be treated chairside (e.g. dental hygiene). Dental recall intervals of 12 months are required for patients who cannot be treated chairside with professional dental hygiene care. However, if they are compliant they will need recall intervals of 12 months augmented by 2–4 oral hygiene sessions. Chairside treatment as conducted in this study can only be achieved if all the caregivers (medical, dental and personal carers) work together to maintain the oral health of patients with special needs. This requires interdisciplinary knowledge, with an understanding of each other’s concerns and limitations, and the financial and human resources to enable collaboration between dentists, anaesthetists, nurses and relatives and caregivers.

### Clinical Relevance

The concept of dental treatment under GA and the following annual dental check-ups have proven themselves in our specialised clinic. In this way, dental emergencies for patients who are difficult to treat have been avoided.
